# An “Outside-In” and “Inside-Out” Consideration of Complement in the Multiple Sclerosis Brain: Lessons From Development and Neurodegenerative Diseases

**DOI:** 10.3389/fncel.2020.600656

**Published:** 2021-01-07

**Authors:** B. Paul Morgan, Jennifer L. Gommerman, Valeria Ramaglia

**Affiliations:** ^1^UK Dementia Research Institute at Cardiff, Cardiff University, Cardiff, United Kingdom; ^2^Department of Immunology, University of Toronto, Toronto, ON, Canada

**Keywords:** complement, multiple sclerosis, pathology, outside-in, inside-out

## Abstract

The last 15 years have seen an explosion of new findings on the role of complement, a major arm of the immune system, in the central nervous system (CNS) compartment including contributions to cell migration, elimination of synapse during development, aberrant synapse pruning in neurologic disorders, damage to nerve cells in autoimmune diseases, and traumatic injury. Activation of the complement system in multiple sclerosis (MS) is typically thought to occur as part of a primary (auto)immune response from the periphery (the outside) against CNS antigens (the inside). However, evidence of local complement production from CNS-resident cells, intracellular complement functions, and the more recently discovered role of early complement components in shaping synaptic circuits in the absence of inflammation opens up the possibility that complement-related sequelae may start and finish within the brain itself. In this review, the complement system will be introduced, followed by evidence that implicates complement in shaping the developing, adult, and normal aging CNS as well as its contribution to pathology in neurodegenerative conditions. Discussion of data supporting “outside-in” vs. “inside-out” roles of complement in MS will be presented, concluded by thoughts on potential approaches to therapies targeting specific elements of the complement system.

## Introduction

Multiple sclerosis (MS) is traditionally considered to be a chronic immune-mediated demyelinating disease of the central nervous system (CNS) mediated by autoreactive lymphocytes that are primed against a CNS antigen in the periphery, enter the CNS *via* breached blood-brain barriers (BBB), and are reactivated in the perivascular spaces of postcapillary venules and meningeal vessels (Engelhardt et al., 2016). As such, one could think of MS as a disease mediated by “outside-in” mechanisms, where the peripheral immune system from the “outside” enters the “inside” of the brain causing injury. Indeed, currently approved disease-modifying treatments (DMT), which are effective in modulating peripheral immunity, reduce demyelinating lesions in relapsing-remitting MS (RRMS). The “outside-in” hypothesis also finds support in genome-wide association studies that identified immune-related susceptibility gene variants in a large cohort of MS patients (International Multiple Sclerosis Genetics Consortium, 2019).

An alternative theory that argues against a causal role of immune cells in the initiation of MS proposes that a primary “cytodegeneration” in the CNS, originating at oligodendrocytes and/or neurons, starts years before the manifestation of any clinical symptoms. In susceptible individuals, this CNS-intrinsic cytodegeneration is followed by an autoimmune inflammatory reaction against antigens that are shed as a result of the primary cellular damage (Stys et al., 2012b). This alternative hypothesis centered on “inside-out” disease mechanisms, finds support in ultrastructural evidence of earliest myelin changes identified in the layer of the myelin sheath that is nearest the axon (Rodriguez and Scheithauer, 1994), in pathological evidence of myelin and axonal degeneration that is not accompanied by evidence of an adaptive immune response in the normal-appearing white matter (Trapp et al., 1998; Henderson et al., 2009), and in clinical evidence of the inability to stop disease progression using immunomodulatory drugs (reviewed in Ciotti and Cross, 2018).

Overall, while inflammatory processes are detected in the CNS of MS patients both at the early and late disease stages (Frischer et al., 2009; Machado-Santos et al., 2018; Fransen et al., 2020) and are undoubtedly important in shaping pathological processes, the evidence is equally consistent with either primary autoimmune pathogenesis of MS (“outside-in” hypothesis) or a model in which an initial injury causes shedding of a high level of autoantigens, which in turn triggers a secondary inflammatory reaction (“inside-out” hypothesis). Therefore, the question remains: is MS initiated by “outside-in” or “inside out” disease mechanisms?

One piece of the puzzle is the complement system, a major arm of the innate immune system that has been implicated in the pathogenesis of MS since the early 1970s (Lumsden, 1971). While it is widely assumed that, in MS, complement proteins derived from the circulation enter the brain parenchyma through a leaky BBB to tag antigen-antibody complexes (“outside-in” paradigm), a potential role of CNS-derived complement components in MS disease processes that originate within the CNS (“inside-out” paradigm) has been largely ignored. This review focuses on how the complement proteins can shape the MS-affected CNS.

## The Complement System

**Figure 1:**
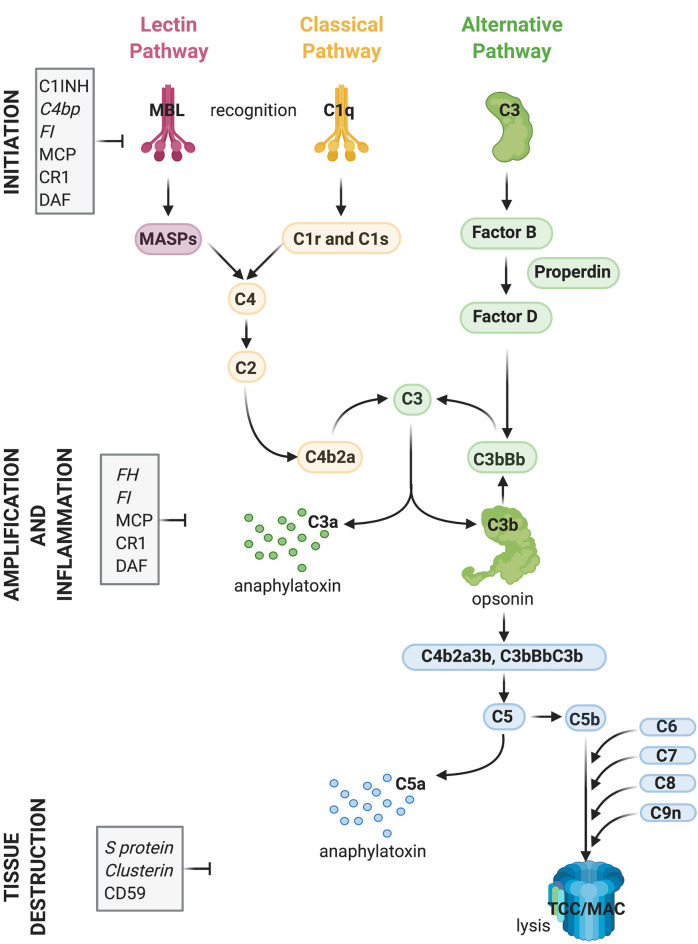
Activation and regulation of the complement (C) system. Recognition of target epitopes by C1q (Classical pathway) or MBL (Lectin pathway) result in the cleavage of C4 and formation of the C3 convertase, cleaving C3. C3 is also activated *via* the alternative pathway by a constant “tick-over” that can be amplified during pathological conditions. Cleaved C3 can nucleate the formation of the C5 convertase, cleaving C5 and eventually forming the terminal complement complex or membrane attack complex (TCC, MAC, C5b9), which can lyse membranes and/or induce cell activation. Activation of C4, C3, and C5 lead also to the formation of anaphylatoxins which cause chemotaxis and inflammation. Activation of the complement system is tightly regulated by soluble (shown in italics) and membrane-bound proteins which can either inhibit the formation or accelerate the decay of the convertases or impede assembly of the C5b9 complex. Abbreviations: C1INH, C1 inhibitor; C4bp, C4 binding protein; FI, factor I; MCP, membrane co-factor protein; CR1, complement receptor 1; DAF, decay-accelerating factor; FH, factor H; MBL, mannose-binding lectin.

Complement plays a central role in the innate immune system. It consists of approximately 50 fluid-phase and cell surface-associated proteins that provide the first line of defense against pathogens and clear immune complexes by tagging and mediating removal of non-self (i.e., pathogens) or altered-self (i.e., dead and dying cells) antigens (Ricklin et al., 2010). Detailed schematics of components and activation products of the complement cascade have been previously published including recently (Morgan and Harris, 2015; Carpanini et al., 2019), and are also shown in Figure 1. The complement system is activated *via* three initiating pathways (classical, alternative, and lectin). Regardless of the initiating pathway, complement activation converges on a common effector pathway, terminating in the formation of the terminal complement complex (TCC) that, in the context of cell membranes, creates a transmembrane pore, the membrane attack complex (MAC). The classical pathway is activated by binding of C1q, a component of the C1 complex, to the Fc domain of antibodies bound to antigens or directly to “danger” epitopes. The lectin pathway is initiated by the binding of mannose-binding lectin to certain carbohydrates expressed on the pathogen surface, whereas the alternative pathway is a powerful amplification loop initiated either spontaneously by low-rate hydrolysis of C3 or by C3b generated in the classical/lectin pathways and progresses through binding of activated factor B and generation of a C3-cleaving enzyme (convertase) on surfaces that lack complement inhibitors (Ricklin et al., 2010).

Regardless of the pathway of activation, complement acts through the production of opsonins (C3b, iC3b, C4b, et cetera) which are molecules that enhance the ability of macrophages and neutrophils expressing complement receptors to phagocytose material; anaphylatoxins (C3a, C5a) which are peptides that induce local and systemic inflammatory responses, increasing the permeability of blood vessels and attracting neutrophils through their chemotactic properties; and through direct killing of organisms by the MAC, which disrupts and forms pores in the phospholipid bilayer of a target cell (Ricklin et al., 2010).

While complement components exert their effector functions against “danger” signals on pathogens, healthy self tissue is protected from undesired complement activation by complement inhibitors present either in the fluid-phase or on membrane surfaces (Hajishengallis et al., 2017). Key complement regulators are indicated in Figure 1. A tight balance between activation and regulation keeps complement in check, whereas disruption of this balance caused by over-activation and/or insufficient regulation can result in tissue injury (Ricklin and Lambris, 2013).

## Sources of Complement Proteins

Although complement components in plasma are synthesized mainly by hepatocytes in the liver, the discovery that CNS-resident cells such as glial cells and neurons can produce complement proteins (Levi-Strauss and Mallat, 1987; Morgan and Gasque, 1997; Gasque et al., 2000), was a prelude to the changing view of immune privilege in the CNS (Engelhardt and Coisne, 2011). For example, it is now appreciated that specialized pockets in the brain are not immune privileged. In particular, the dura that lies above the leptomeninges is transversed by functional lymphatics and fenestrated vasculature that lacks tight junctions, making it highly permeable to peripheral immune cells and soluble molecules (including complement proteins) that enter into the dura, even at a steady-state (reviewed in Rua and McGavern, 2018; Ramaglia et al., in press b). Therefore, in addition to a parenchymal source of complement, serum-derived complement can enter the brain either through a leaky blood-brain barrier (BBB) and/or through a normally highly permeable meningeal brain barrier.

While little is known about the synthesis of terminal complement components in the brain (or whether this occurs at all), C1q and C3 synthesis in the brain has been studied in more detail. Astrocytes have been shown to produce C3 and Factor B, components of the alternative complement pathway, *in vitro* (Levi-Strauss and Mallat, 1987). Cultured human microglia were also shown to express C1q, C3, and C4 (Walker et al., 1995). Glioma cell lines have also been a useful tool to study complement biosynthesis by glial cells and have shown to be able to produce proteins of the complement alternative pathway, namely C3, factor B, factor H, and factor I (Gasque et al., 1992) and proteins of the classical complement pathway, namely C1q, C1r, C1 s, C1-Inh, C2, C4, and C5 (Gasque et al., 1993). Astrocytes and microglia also express receptors for the complement activation products C3d and C3a (Gasque et al., 1996) and C5a (Gasque et al., 1995, 1997). In addition to glial cells, also neuronal cell lines can express complement components and regulators (i.e., C3, factor H (FH), factor B (FB), C4, C1-inhibitor (C1-inh), C1q, C5, C6, C7, and C9; Thomas et al., 2000). Early studies have also shown local production of complement proteins in the nervous system *in vivo* in response to peripheral nerve injury (Svensson and Aldskogius, 1992) or experimental lesions in the rat brain (Pasinetti et al., 1992).

More recent studies have shown that during the development of the visual system, synthesis of C1q is upregulated in neurons (Stevens et al., 2007). C1q mRNA and protein expression are also dramatically increased during normal aging of the mouse and human brain (Stephan et al., 2013; see “Early Complement Components in the Developing, Adult, and Normal Aging CNS” section). Using *in situ* hybridization, western blot, and immunohistochemical analysis of the myelinated and demyelinated MS hippocampus compared to controls, Michailidou and colleagues showed that C1q is synthesized also by hippocampal neurons and its expression increases intracellularly in neurons within MS tissue (Michailidou et al., 2015). A subsequent study showed that neuronal C1q synthesis, identified by *in situ* hybridization, occurs also in other areas of cortical gray matter in the MS brain (Watkins et al., 2016; see “Evidence Supporting the ‘Outside-In’ Paradigm” section). However, one study contradicting the concept that neurons represent an important source of C1q showed that microglial-specific deletion of C1q in mice results in lack of C1q in the adult brain while blood C1q levels remain unchanged, implicating microglia as the predominant CNS source of C1q (Fonseca et al., 2017).

In terms of C3, its synthesis has been reported in reactive astrocytes in MS and other neurodegenerative diseases, with C3 now being considered a marker for the designation of “A1” neurotoxic astrocytes (Liddelow et al., 2017). A1 astrocytes lose their ability to promote neuronal survival, outgrowth, and synaptogenesis, and instead induce the death of neurons and oligodendrocytes (Liddelow et al., 2017). Another study has shown neuronal synthesis of C3 in advanced MS cases. These C3-producing neurons were observed close to C3d^+^ microglial clusters at the edge of slowly expanding lesions (see “Evidence Supporting the ‘Inside-Out’ Paradigm” section; Michailidou et al., 2017). Altogether these data show that early complement components, particularly C1q and C3, are present in both the developing brain and normal aging brain independent of the breakdown of the blood-brain barrier, while the synthesis of complement proteins by neurons and glia is increased in the diseased brain, including in MS.

## Early Complement Components in the Developing, Adult, and Normal Aging CNS

**Figure 2:**
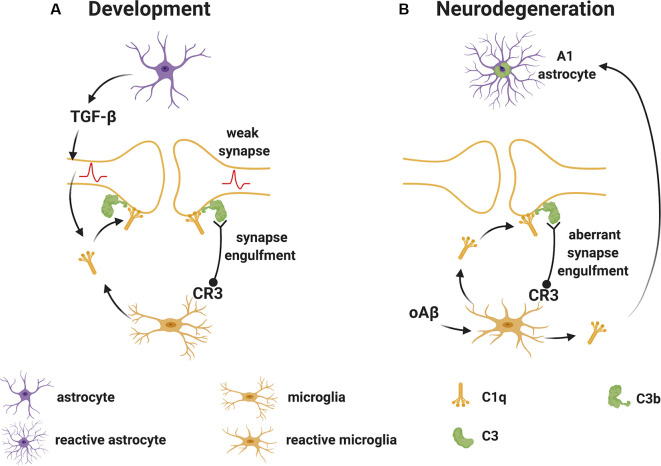
Complement-mediated synapse elimination during development and in neurodegenerative diseases.** (A)** In the developing brain, astrocytes produce TGF-β that promotes the production of C1q by neurons. Neuron-derived and microglia-derived C1q tags weak synapses activating the classical complement pathway which results in the cleavage of C3 with deposition of C3b at synapses. C3b-tagged synapses are then removed through phagocytosis by microglia expressing the complement receptor CR3. **(B)** In neurodegenerative diseases, such as Alzheimer’s disease (AD), oAβ promotes the production of C1q by microglia. C1q-tagged synapses are then eliminated by microglia *via* the classical complement pathway as observed for synapses during development. Also, microglia-derived C1q (together with IL-1α and TNF) induces a subtype of C3^+^ reactive astrocytes, termed A1 astrocytes, which are neurotoxic. Abbreviations: oAβ, oligomeric amyloid β; TGFβ, transforming growth factor β; IL, interleukin; TNF, tumor necrosis factor.

Stevens et al. (2007) were the first to demonstrate a substantial role for the upstream “early” classical complement pathway (C1q and C3) in eliminating redundant synapses during the development of the retinogeniculate system in the visual thalamus. In this system, extranumerary synapses are targeted by the complement component C1q, opsonized by C3 and phagocytosed by microglia *via* the complement receptor CR3 (Schafer et al., 2012), in a process which appears to be independent of the formation of the terminal “late” activation effector TCC/MAC (Figure 2A). This is supported by experimental studies in mice, where deletion of C1q, C3 or CR3, or pharmacological (minocycline) disruption of microglia-mediated engulfment of synapses during early development leads to defects in eye-specific segregation of retinal ganglion cell projections (Stevens et al., 2007; Schafer et al., 2012). Therefore, the classical complement cascade is involved in mechanisms of synaptic refinement, similarly to what has been previously described for other molecules. These include pattern recognition molecules such as pentraxins (Bjartmar et al., 2006), the Triggering Receptor Expressed on Myeloid Cells 2 (TREM2; Filipello et al., 2018), and the Class I Major Histocompatibility Complex. Involvement of the latter has been shown using mice in which deletion of two of the MHCI genes [H2-K(b) and H2-D(b)], impairs developmental refinement of retinogeniculate projections (Corriveau et al., 1998; Datwani et al., 2009).

During normal development, the complement components that participate in the pruning of synapses are likely produced locally in the brain since the BBB protects the CNS parenchyma from plasma-derived complement and immune cells. As mentioned, C1q can be expressed by microglia and certain neurons such as the retinal ganglion cells (RGC) in the visual system (Stevens et al., 2007). The cytokine transforming growth factor-β (TGF-β) has been identified as a factor secreted by astrocytes that is necessary and sufficient for C1q expression in purified RGC (Bialas and Stevens, 2013). *In vivo*, TGF-β receptors (TGFβRII) are expressed in RGC during development and specific disruption of TGFβRII in retinal neurons significantly reduces C1q expression, decreases synaptic localization of C1q, and inhibits synaptic pruning in the visual thalamus (Bialas and Stevens, 2013). Other cytokines also play roles in synapse pruning during development. For example, the interleukin-1 family cytokine interleukin-33 (IL-33) orchestrates astrocyte-microglial communication to tune microglial engulfment of synapses (Vainchtein et al., 2018). IL-33 is produced by synapse-associated astrocytes and signals to microglia to promote increased synaptic engulfment; deletion of IL-33 leads to excess synapses and abnormal thalamic and sensorimotor (spinal cord) circuit function, demonstrating the requirement for IL-33 in synaptic development in the thalamus and spinal cord (Vainchtein et al., 2018).

The difference between synapses that are tagged for elimination by complement vs. those that are spared is a key question. Certain cell surface markers are implicated as discriminating signals that direct the sparing of synapses. For example, a recent study demonstrated that CD47, the high-affinity receptor for thrombospondin-1, is required to prevent excess pruning of retinogeniculate synapses during development (Lehrman et al., 2018). CD47 is a “don’t eat me” signal and self-associated molecular pattern (SAMP) that is expressed on many cell types throughout the body (Reinhold et al., 1995), and is also found on synapses (Mi et al., 2000). It can directly inhibit phagocytosis by binding the signaling molecule SIRPα, on macrophages and other professional phagocytes (Oldenborg et al., 2000; Okazawa et al., 2005). CD47 can also prevent phagocytosis of cells that are opsonized with “eat me” flags, such as complement, showing that it can overrule these signals (Oldenborg et al., 2001). Interestingly, CD47 was shown to preferentially localize at more electrically active synapses in wild-type mice (Lehrman et al., 2018), pointing to the sparing of “stronger” or more active inputs. In line with this finding, CD47 knockout mice did not show the expected preferential microglial engulfment of less active inputs, and both CD47 knockout and SIRPα knockout mice displayed excess microglial engulfment of retinal ganglion cell synaptic inputs (Lehrman et al., 2018). Together, these data suggest that CD47 expression protects certain (potentially more active) synaptic populations from targeting by microglia, while CD47^low^ synapses may be preferentially targeted for removal. Another mechanism potentially regulating the process of C1q-mediated elimination of synapses involves apoptosis. Proteomic investigation and pathway analysis of C1q-tagged synaptosomes revealed the presence of apoptotic molecules (Vdac1, Prdx6, Uchl1, Eno1, Ppp3r1, and Nptx1) in C1q-tagged synapses (Györffy et al., 2018), suggesting that synaptic pruning may involve some of the same molecular triggers that normally stimulate the homeostatic (non-phlogistic) process of complement-mediated enhanced clearance of apoptotic cells in the periphery (Ricklin et al., 2010).

Pruning of excessive synapses does not only occur during postnatal CNS development but is also involved in the dynamic remodeling of synapses which occurs constantly in mature neurons throughout life as a result of experience and learning (Trachtenberg et al., 2002; Tropea et al., 2010; Fu and Zuo, 2011). A recent study showed that the C1q-dependent classical complement pathway is actively involved in synapse elimination by microglia in the healthy adult hippocampus (Wang et al., 2020). C1q was found to localize to synapses and dendritic spines of engram cells, the neurons responsible for the storage of memory in the hippocampus. The identification of C1q-tagged synaptic components within the lysosomes of microglial cells and the finding that depleting microglia or inhibiting microglia-mediated phagocytosis prevented loss of synapses and memory impairment, indicated that microglia are responsible for the elimination of synapses in the healthy brain and that complement provides the relevant phagocytic signal. Also, specific overexpression of the complement regulator CD55 in engram cells protected from memory loss, further indicating that the elimination of synaptic elements by microglia in the healthy adult hippocampus occurs in a complement-dependent manner. In contrast, inhibiting the activity of engram cells exacerbates memory loss, and this could be blocked by depleting microglia or inhibiting complement pathways in engram cells (Wang et al., 2020). These data indicate that synapse elimination by microglia in the adult brain is also activity-dependent, following similar rules to those in the developing brain (Schafer et al., 2012), thus resulting in the erasure of less-active synapses.

Finally, C1q-dependent reorganization of brain circuitry also occurs during normal aging. C1q levels in the brain dramatically increase during aging (by as much as 300-fold in 24 months old mice compared to early postnatal levels and by 8-fold in hippocampi from 75–77-year-old humans compared to 1–2 months old infants) and C1q can be found close to synapses. Importantly, C1q-deficient mice exhibit enhanced synaptic plasticity and less cognitive and memory decline when aged (Stephan et al., 2013).

## Early Complement Components in Neurodegeneration

Given that complement plays a key role in developmental synapse elimination, an intriguing hypothesis is that these normal developmental mechanisms may become reactivated during neurodegeneration, driving pathology in settings such as Alzheimer’s disease (AD) and Prion disease. Indeed, in AD and prion disease, neuronal death is preceded by synaptic dysfunction and loss (Schneyer and Hall, 1975; Mallucci, 2009). If synaptic alterations indeed precede and predict neuronal death, then early targeting of the pathways responsible for synaptic injury would be an obvious therapeutic approach to prevent loss of neurons. Since the involvement of key complement factors in neurodegeneration has been previously reviewed (Bonifati and Kishore, 2007; Brennan et al., 2016; Carpanini et al., 2019; Lee et al., 2019) including recently in AD (Tenner, 2020), this section will focus on the emerging evidence that supports a role for complement activation in driving loss of synapses early in the disease.

The initial evidence implicating classical complement pathway components in synaptic loss during neurodegeneration comes from studies in glaucoma, a disease characterized by elevation of intraocular pressure, loss of RGC neurons, and degeneration of the optic nerve, eventually resulting in blindness (Williams et al., 2016). Expression profiling approaches in glaucomatous DBA/2J mice that develop pathology closely resembling the human disease showed that the expression of classical complement components is upregulated in the mouse retina during early glaucoma stages, before detectable signs of neurodegeneration (Steele et al., 2006; Howell G. R. et al., 2011). Furthermore, C1q immunoreactivity was upregulated in the layer of the retina enriched in synapses, specifically post-synaptic connections of RGCs, and increased C1q expression was temporally correlated with a decrease in synaptic density (Stevens et al., 2007). Importantly, the most compelling evidence that complement-mediated elimination of synapses may be an early and injurious event in glaucoma is the demonstration that either deletion of C1q in DBA/2J mice or C1 inhibition in a rat glaucoma model confers neuroprotection in the glaucomatous eye (Howell G. R. et al., 2011; Williams et al., 2016).

As mentioned, the complement system has also been implicated in the pathology of the AD brain where activation of virtually all complement components and activation products have been detected (Veerhuis et al., 2011). In terms of early complement proteins, C1q, C3b, C4b, and properdin have all been localized to key pathological hallmarks of AD, such as amyloid plaques and neurofibrillary tangles (hyperphosphorylated τ) in human AD and animal models, supporting the activation of both classical and alternative pathways *in vivo* (reviewed in Fonseca et al., 2011; Veerhuis, 2011). In this context, complement activation may be beneficial through the opsonization and clearance of misfolded proteins (Maier et al., 2008; Fu et al., 2012). Also, C1q has been shown to promote the clearance of apoptotic neurons and neuronal blebs in the AD brain (Fraser et al., 2010). Importantly, sites of protein aggregates and dead cells are decorated with membrane-bound and soluble complement regulators, such as CD55, Factor H, and C4 binding protein (C4BP; Strohmeyer et al., 2002; Trouw et al., 2008; Martin and Blom, 2016), demonstrating an orchestrated mechanism of a complement-mediated process comprising activation that is locally controlled to allow clearance of targeted tissue while limiting activation downstream of the C3 and C5 convertases and the associated pro-inflammatory activities.

Studies in mouse models of AD have helped our understanding of the roles and consequences of early complement activation in AD pathology. For example, human amyloid precursor protein (hAPP) transgenic mice deficient in C3 or overexpressing the C3 convertase inhibitor complement receptor 1-related protein y (Crry), showed greater amyloid accumulation and greater cognitive deficits than control mice, suggesting a protective role for C3 activation (Wyss-Coray et al., 2002; Maier et al., 2008). In contrast, C1q deletion in the Tg2576-transgenic mouse model of AD resulted in diminished plaque burden, a reduced loss of hippocampal synapses, and less cognitive decline relative to C1q sufficient Tg2576 mice, suggesting a detrimental role for C1q in the model (Fonseca et al., 2004). Also, microglia-derived C1q (together with IL-1α and TNF) induced astrocytes to adopt a neurotoxic A1 phenotype (Liddelow et al., 2017). In more recent studies in other AD models, C1q was found to be increased and deposited at synapses before overt plaque deposition, whereas deletion of C1q or C3 or the complement receptor CR3 (also expressed on microglia), reduced the number of phagocytic microglia as well as the extent of early synapse loss, resulting in improved cognitive function (Hong et al., 2016; Figure 2B). Similar evidence of the involvement of early complement components in pathological synapse elimination has been reported in models of frontal temporal dementia (Lui et al., 2016) and West Nile virus infection (Vasek et al., 2016).

Loss of synapses and upregulation of complement components are also major events in other neurodegenerative diseases including Huntington’s disease (Moller, 2010) and Parkinson’s disease (Chao et al., 2014), however, it is unclear whether in these conditions complement plays a crucial role in the elimination of synapses.

## A Link Between Inflammation and Injury in the MS-Affected CNS

The past decade has produced a wealth of studies aimed at understanding the link between inflammation and CNS injury in MS, especially in the later disease stages. Most patients present with RRMS, but the disease eventually transitions into a progressive form (secondary progressive multiple sclerosis, SPMS), and a minority of patients develop progressive disease from the onset (primary progressive multiple sclerosis, PPMS; Lublin et al., 2014). While DMT have been successful in controlling the RRMS form of the disease, they, unfortunately, perform poorly in preventing disease progression and accumulation of clinical disability in PPMS and SPMS patients (Macaron and Ontaneda, 2019). If on one hand, these clinical observations have validated a key role of inflammation from the “outside” in the pathogenesis of RRMS, on the other hand, they have raised doubts regarding whether similar mechanisms are at play during the progressive stage of the disease. The reason DMT fail in SPMS and PPMS may be that the inflammatory targets of these DMT are not relevant in progressive forms of MS. Alternatively, inflammation may still be involved during the progressive phase of the disease, but such inflammatory processes become increasingly compartmentalized within the CNS behind a relatively intact blood-brain barrier (Kutzelnigg and Lassmann, 2014). This hypothesis is supported by pathological (Serafini et al., 2004; Lassmann et al., 2007; Magliozzi et al., 2007, 2010; Howell O. W. et al., 2011; Howell et al., 2015; Lucchinetti et al., 2011; Popescu et al., 2011; Choi et al., 2012; Haider et al., 2016) and imaging studies (Absinta et al., 2015; Zurawski et al., 2020) which—except for some studies (Kooi et al., 2009; Ighani et al., 2020)—have identified aggregates of immune cells in the meninges of MS patients overlaying areas of cortical injury. While these studies implicate inflammatory events within the CNS compartment, this theory still puts the immune system upstream of the disease mechanisms in MS (“outside-in” hypothesis; Kutzelnigg and Lassmann, 2014). Overall, it is now widely accepted that inflammation persists and is linked to tissue injury in both white and gray matter tissue of patients with progressive disease (Magliozzi et al., 2007, 2010; Frischer et al., 2009; Howell O. W. et al., 2011; Machado-Santos et al., 2018); however, the evidence is equally consistent with either the “outside-in” or the “inside-out” paradigm (Stys et al., 2012b). In this section, we present the evidence linking key features of progressive MS white and gray matter pathology with inflammation and specifically complement activation.

### MS White Matter

**Figure 3:**
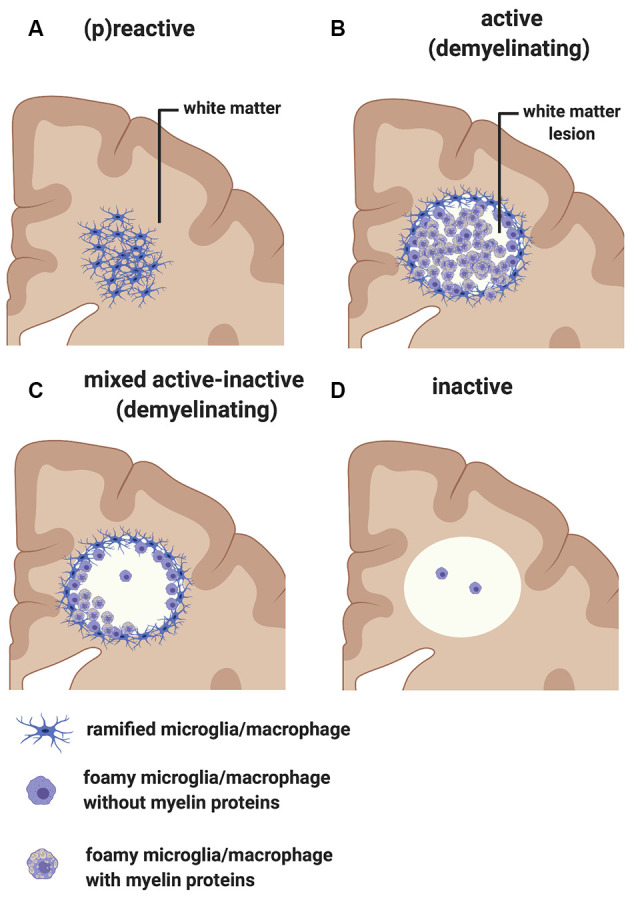
Classification of white matter lesions in multiple sclerosis (MS). Schematic diagram illustrating different types of MS white matter lesions, including **(A)** (p)reactive sites, **(B)** active demyelinating lesions, **(C)** mixed active-inactive demyelinating lesions, and **(D)** inactive lesions. See the text in “MS White Matter” section for a detailed description of each lesion type.

Early pathological studies had already demonstrated that focal demyelinated lesions in the white matter are generally centered on large or medium-sized veins (Rindfleisch, 1863), pointing to a link between events in the periphery and white matter injury. Follow up studies have further highlighted the relationship between inflammation and white matter pathology by showing that lymphocytes and monocytes/macrophages accumulate in the perivascular spaces of medium-sized and small veins within MS plaques. These inflammatory cells can also infiltrate the tissue parenchyma, while local microglial cells become activated (Charcot, 1880). In addition to the lesions, unlesioned areas of the MS-affected CNS also undergo changes that prelude further injury. The so-called “normal-appearing white matter” is characterized by perivascular inflammatory infiltrates, astrocytic gliosis, diffuse axonal injury, and diffuse microglial activation that increases with longer disease duration (Kutzelnigg et al., 2005)—thus, not so normal. Also, within unlesioned tissue, aggregates of HLA^+^ microglia/macrophages are observed in proximity to microvessels surrounded by lymphocytic infiltrates identifying “(p)reactive lesions” (Figure 3A), so-called since they may or may not represent the earliest stages in the formation of plaques (Barnett and Prineas, 2004; Marik et al., 2007). Active lesions are highly inflammatory and are found most frequently in RRMS (Figure 3B). They are characterized by loss of myelin, with HLA^+^ and CD68^+^ microglia/macrophages infiltrating across the lesion. Foamy microglia/macrophages within an MS lesion indicate ongoing phagocytic activity and myelin proteins are detected within the cytoplasm of these myeloid cells (Lucchinetti et al., 2000; Kuhlmann et al., 2017). Histological studies using newly identified markers selectively expressed on microglia, including TMEM119 which differentiates yolk sac-derived CNS-resident microglia from bone marrow-derived recruited monocytes/macrophages, and the purinergic receptor P2RY12 which identifies a homeostatic microglia phenotype (Butovsky et al., 2014; Masuda et al., 2020), showed that ~45% of macrophage-like cells are derived from the resident microglia pool in active lesions (Zrzavy et al., 2017). In these lesions, microglia show reduced expression of the homeostatic marker P2RY12, which is reacquired in inactive lesions. T cells and CD20^+^ B cells are also detected within active lesions (Machado-Santos et al., 2018). While B cells in the lesion are mostly seen in the perivascular space of the central vein, T cells infiltrate the parenchyma in addition to populating the perivascular space. Some CD8^+^ T cells feature a more tissue-resident memory phenotype (i.e., CD8α/α, CD103^+^; Machado-Santos et al., 2018). Tissue-resident memory T cells lose expression of surface molecules that are involved in the egress of leukocytes from inflamed tissue (S1P1 or CCR7). These molecular changes have been suggested as a potential mechanism responsible for the compartmentalized inflammatory response in MS lesions (Machado-Santos et al., 2018). In addition to CD8^+^ T cells, CD4^+^ T cells are also found in MS lesions and have been shown to produce cytokines such as IL-17 and IL-22 which possibly bind to BBB endothelial cells expressing IL-17 and IL-22 receptors, thereby gaining access to the CNS parenchyma (Kebir et al., 2007). Plasma cells are also detected within MS lesions but their phenotype is less well understood (Machado-Santos et al., 2018). Over time, active lesions develop into mixed active/inactive lesions (Figure 3C). Indeed, these types of lesions are most commonly found in patients with advanced (disease duration of more than 10 years) or SPMS disease course. They are characterized by a border of activated HLA^+^ and CD68^+^ microglia/macrophages and a hypocellular core. In a subset of these lesions, called slowly expanding or “smoldering”, microglia/macrophages at the rim contain MBP or PLP myelin degradation products in their cytoplasm, indicating either a new wave of inflammation and demyelination, or documenting the last remnant of an earlier demyelinating lesion (Frischer et al., 2015). With time, active and mixed active/inactive lesions transition into inactive lesions (Figure 3D). These are indeed the dominant plaque type in patients with advanced disease (duration of more than 15 years) or SPMS without attacks (Frischer et al., 2015). Inactive lesions are demyelinated but hypocellular throughout (Frischer et al., 2015).

Overall the pathology of the white matter shows that inflammation is very much linked to demyelination in the MS brain, but it does not answer whether inflammation is the primary cause of the injury (consistent with the “outside-in” paradigm) or a secondary reaction (consistent with the “inside-out” paradigm).

### MS Gray Matter

Although gray matter changes in the MS brain were noted early on (Dawson, 1916; Brownell and Hughes, 1962), it was not until the advent of more sensitive imaging and histology techniques that the true extent of gray matter demyelination was appreciated, reaching up to 90% of the cortex in extreme cases (Kidd et al., 1999; Peterson et al., 2001; Bø et al., 2003; Chard and Miller, 2009; Calabrese et al., 2010). Based on the location of the demyelinated area, different types of gray matter lesions have been identified (Bø et al., 2003; Kuhlmann et al., 2017). Subpial lesions, occurring on the surface of the brain and often affecting several adjacent gyri are perhaps the most intriguing since they are unique to MS and not seen in any other neuroinflammatory diseases (Fischer et al., 2013). Therefore, they could hold the key to MS-specific disease mechanisms. Subpial cortical demyelination, although present in earlier stages of MS (Lucchinetti et al., 2011; Popescu et al., 2011), appears to be more dominant in the later progressive stage of the disease (Peterson et al., 2001; Bø et al., 2003; Kutzelnigg et al., 2005). Importantly, subpial changes are not limited to demyelination but also include neuronal (Magliozzi et al., 2010), axonal (Magliozzi et al., 2007), and synaptic (Wegner et al., 2006; Dutta et al., 2011) injury with the extent of subpial cortical damage now considered to be a major contributor to disease progression in MS including both physical (Harrison et al., 2015) and cognitive (Calabrese et al., 2012) impairments. For example, the hippocampus, which is the portion of the cortex critically important for cognitive functions such as the formation, consolidation, and recollection of memories (Squire et al., 2004), is profoundly affected in MS. Not only is the hippocampus extensively demyelinated (Geurts et al., 2007), but it also shows evidence of neuronal injury (Papadopoulos et al., 2009) and synaptic abnormalities (Dutta et al., 2011). Structural and functional disconnections of the hippocampus from several brain networks have also been revealed by magnetic resonance imaging (MRI) studies (Sicotte et al., 2008; Gold et al., 2010, 2014; Longoni et al., 2015). Experimental (Trapp et al., 2007; Werneburg et al., 2020) and post-mortem (Peterson et al., 2001; Werneburg et al., 2020) studies indicate that disconnection of brain networks in MS may be mediated by the loss of synapses *via* the pruning process, part of which is complement-mediated (see “Evidence Supporting the ‘Inside-Out’ Paradigm” section).

Whether inflammation causes subpial cortical gray matter damage has been debated. When compared with the highly inflammatory features of white matter lesions described above, subpial gray matter lesions are relatively “non-inflammatory” in nature. They lack evidence of major BBB disturbances (van Horssen et al., 2007) and display a paucity of parenchymal immune cell infiltration (Peterson et al., 2001; Bø et al., 2003). Also, current anti-inflammatory treatments that effectively modulate peripheral immunity do not prevent or resolve gray matter damage (Ciotti and Cross, 2018).

One theory supporting a key role of inflammation in subpial cortical pathology, while reconciling pathological and clinical findings of progressive MS, proposes that this injury may be driven by a compartmentalized immune response involving the inflamed leptomeninges within brains that have a relatively intact BBB (Serafini et al., 2004; Lassmann et al., 2007; Magliozzi et al., 2007, 2010; Howell O. W. et al., 2011; Lucchinetti et al., 2011; Popescu et al., 2011; Choi et al., 2012; Absinta et al., 2015; Howell et al., 2015; Haider et al., 2016). Histological examination of autopsy CNS tissue from progressive MS cases have shown evidence of Tertiary Lymphoid Tissues (TLT) in the leptomeninges lining the forebrain (Serafini et al., 2004; Magliozzi et al., 2007), the cerebellum (Howell et al., 2015) and to a lesser extent the spinal cord (Reali et al., 2020). MRI studies have also confirmed the presence of meningeal TLT in the MS brain (Absinta et al., 2015). Importantly, these leptomeningeal TLT are associated with underlying (subpial pattern) demyelination, neuronal loss, and a gradient of microglial activation from the subpial area moving inward into the tissue (Magliozzi et al., 2010; Howell O. W. et al., 2011). The meningeal TLTs harbor B cells, T cells, dendritic cells, macrophages, monocytes, plasma cells, and stromal cells that resemble Follicular Dendritic Cells (FDC) normally found in germinal centers (Serafini et al., 2004; Magliozzi et al., 2007; Howell O. W. et al., 2011; Lucchinetti et al., 2011; Lagumersindez-Denis et al., 2017; Reali et al., 2020). While germinal center environments can support the secondary diversification of B cell receptors (affinity maturation and class switch) in the context of some animal models of MS (Galicia et al., 2013), a study using post-mortem samples suggested that the majority of these secondary diversification events are occurring within the draining cervical lymph node rather than in the CNS itself (Stern et al., 2014). Nonetheless, it has been shown that within TLT, B cells proliferate and CD21^+^ CD35^+^ FDC-like stromal cells produce the chemokine CXCL13, suggestive of an immunocompetent stromal cell niche that serves to attract and retain leukocytes, supporting their survival and proliferation (Serafini et al., 2004).

An important function of cells that reside within the meninges is the ability to secrete by-products which are released into the CSF and may diffuse freely throughout the subarachnoid space. If one considers that there are regions of reduced CSF flow, particularly within brain sulci, such by-products may become concentrated in “hot spots,” resulting in the trapping of immune cells (Howell O. W. et al., 2011). These soluble molecules are potentially inflammatory, cytotoxic, myelinotoxic but also potentially anti-inflammatory and neuroprotective. Indeed, recent studies analyzing the content of CSF in MS patients have shown that high cortical lesion load correlates with proinflammatory cytokines (IFNγ, TNF, IL2, and IL22; Gardner et al., 2013; Magliozzi et al., 2018), molecules related to sustained B-cell activity and lymphoid-neogenesis (CXCL13, CXCL10, LTα, IL6; Magliozzi et al., 2018), B-cell survival factors (BAFF; Magliozzi et al., 2019), factors indicative of BBB leakage (fibrin, complement and coagulation factors; Magliozzi et al., 2019) and iron-related proteins (free-hemoglobin and haptoglobin; Magliozzi et al., 2019), but also anti-inflammatory mediators such as IL10 (Magliozzi et al., 2019). In line with the latter, regulatory T (Treg) cells producing IL-10 have been detected in the MS CSF (Feger et al., 2007) although deficits of their regulatory functions and migratory properties have also been reported in MS (Viglietta et al., 2004; Astier et al., 2006; Martinez-Forero et al., 2008; Venken et al., 2008; Schneider-Hohendorf et al., 2010).

In summary, while the spatial association between TLT and subpial injury has suggested a potential role in initiating and/or modulating demyelinating pathology in the gray matter, these same pathological features are equally consistent with a potential reaction of the immune system in the meningeal compartment to areas of pre-existing injury in the underlying cortex. Without longitudinal studies, it is impossible to conclude whether these TLT sites preceed the formation of subpial lesions, or whether they represent an attempt of the immune system to repair an existing injury. If the latter scenario is true, then the presence of meningeal TLT reacting to a site of primary injury would be consistent with the “inside-out” paradigm. Studies in animal models that replicate meningeal TLTs and subpial pathology (Pikor et al., 2015; Ward et al., 2020), emerging MRI approaches to identify and perhaps monitor the formation of TLT in patients (Absinta et al., 2015), and multi-dimensional technologies to phenotype cells *in situ* (Ramaglia et al., 2019) will be important to unravel the nature of the relationship between TLTs and subpial injury in MS.

## Complement in MS

A contribution of the complement system to the pathology of MS has long been suspected based on pathological and serological studies in MS as well as functional studies in EAE. However, the extent and trigger(s) of complement activation and the pathways involved in specific disease processes remain unclear. In particular, the relative contribution of serum-derived complement vs. CNS-derived complement in initiating as well as propagating MS disease processes remains to be determined. In this section, the evidence is reviewed that supports a role for complement in both “outside-in” and “inside-out” pathological mechanisms of the MS-affected CNS.

### Evidence Supporting the “Outside-In” Paradigm

**Figure 4:**
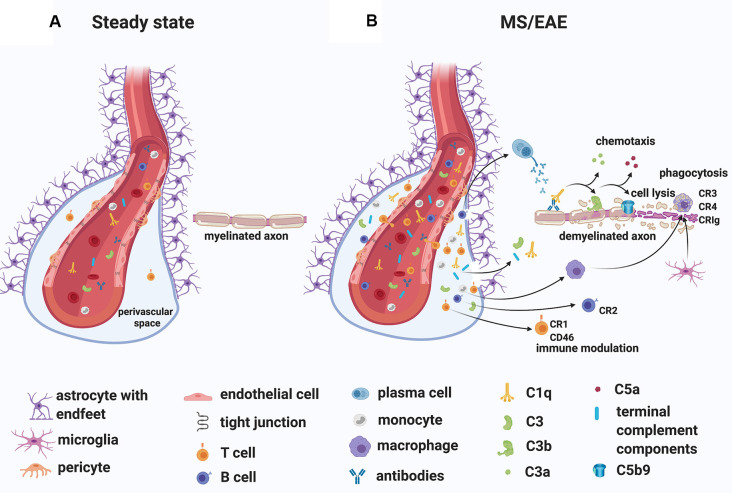
A role for complement in the “outside-in” paradigm of white matter demyelination in MS.** (A)** During steady state, complement components circulate in the blood. Only leukocyte extravasation across the blood-brain barrier (BBB) occurs but is limited to few activated CD4^+^ T lymphocytes that perform immunological surveillance. These activated lymphocytes interact with adhesion molecules (ICAM-1, VCAM-1) expressed on the lumen of vascular endothelial cells. Chemokine (CXCL12) expression by endothelial cells on the abluminal side contributes to sequestering the activated CD4^+^ T cells in the perivascular space through binding to the chemokine receptor (CXCR4) on the T cell. **(B)** During MS/EAE, the efficiency of leukocyte diapedesis is increased. CCL19, CCL21, and CXCL12 are up-regulated by cerebrovascular endothelial cells that promote the recruitment and adhesion of CD4^+^ T cells primed in the periphery against a CNS antigen. On the abluminal surface of endothelial cells, CXCR7 binds to CXCL12 to reduce T cell sequestration in the perivascular space. Within the perivascular space, activated T cells produce chemokines and cytokines (such as TNFα and GM-CSF) that promote the recruitment of myeloid cells from the blood. Matrix metalloproteinases (MMP-2 and MMP-9) are produced and selectively cleave dystroglycan in the astrocytic foot processes, allowing penetration of T cells, B cells, monocytes, and complement into the CNS parenchyma. Within the CNS, encephalitogenic T cells re-encounter their specific antigen and are re-activated, producing inflammatory cytokines. T cells can also bind directly to myelin epitopes producing cytotoxic mediators and leading to activation of macrophages. Infiltrating B cells transition into antibody-producing plasma cells perhaps *in situ*. Autoantibodies can also enter the CNS through a breached BBB. Complement components enter the CNS *via* a breached BBB and are activated either by the recognition of antigen/antibody complexes or directly by “altered self” myelin epitopes. Complement-tagged myelin is then phagocytosed by phagocytes expressing complement receptors (macrophages and microglia). Activated complement components can also bind T cells and B cells *via* their complement receptor modulating their function (see details in text). Abbreviations: CNS, central nervous system; TNFα, tumor necrosis factor α; GM-CSF, granulocyte-macrophage colony-stimulating factor; CR, complement receptor.

While the specific antigens responsible for initiating the immune response in MS remain unidentified, MS patients commonly have oligoclonal immunoglobulin G in the CSF (Joseph et al., 2009) and several studies have demonstrated antibodies to myelin and other CNS autoantigens in patients (reviewed in Berger and Reindl, 2007). Moreover, T cells specific for myelin antigens have been detected in the blood of MS patients (reviewed in Kaskow and Baecher-Allan, 2018) and found to produce inflammatory mediators such as granulocyte-macrophage colony-stimulating factor (GM-CSF), tumor necrosis factor (TNF), interferon-gamma (IFN-γ), interleukin-2 (IL-2) and C-X-C chemokine receptor type 4 (CXCR4; Galli et al., 2019). Importantly, transient gadolinium-enhancing lesions on magnetic resonance scans have indicated BBB breakdown, especially in RRMS patients (Stone et al., 1995). In line with this observation, immunoglobulins and complement deposits have been described in MS lesions (Compston et al., 1989; Storch et al., 1998; Lucchinetti et al., 2000; Prineas et al., 2001; Ingram et al., 2014; Michailidou et al., 2015, 2017; Watkins et al., 2016; Loveless et al., 2018). Also, plasma proteins including Factor XIIa, plasmin, and thrombin, which are part of the coagulation pathway, could leak from the circulation into the brain parenchyma through a compromised BBB, particularly in RRMS patients, triggering direct activation of C3 (Markiewski et al., 2007; Amara et al., 2010) and C5 (Huber-Lang et al., 2006). Notably, *in situ* evidence of antibody and complement deposits within MS plaques in close association with capillary endothelial cells (Compston et al., 1989; Storch et al., 1998), have suggested a role for an “outside-in” complement/antibody-mediated injury in lesions (Figure 4). Moreover, complement components and regulators are elevated systemically and in the CSF of MS patients (Ingram et al., 2009; Ingram et al., 2010a, b; Ingram et al., 2012; Zelek et al., 2019) and early complement pathway gene variants (C3, C1QA, and CR1) have recently been associated with structural and functional measures of MS severity (Fitzgerald et al., 2019), indicating an inherent susceptibility to complement-mediated injury in some patients.

A detailed pathological analysis performed by Lucchinetti et al. (2000) on active and early demyelinating MS white matter lesions from a cohort of patients with relatively short disease duration (the mean disease duration before autopsy or biopsy was 39 and 9 months, respectively), identified a histological pattern (pattern II) of lesions with prominent complement activation in a distinct but large subgroup (pattern I: 4%, pattern II: 59%, pattern III: 26%, pattern IV: 11% of total autopsy cases analyzed) of MS patients. In these lesions, the terminal complement TCC/MAC was associated with phagocytic macrophages. These findings suggested that complement activation may be one of at least four possible mechanisms of tissue injury leading to demyelination. Other patterns involve activated macrophages/microglia (but not complement activation; pattern I), apoptotic (pattern III), or non-apoptotic (pattern IV) oligodendrocyte degeneration (Lucchinetti et al., 2000). While this study highlighted the heterogeneity of early MS lesions, a subsequent report by Breij et al. (2008) based on the analysis of white matter lesions in autopsy tissue from MS patients with long disease duration (mean disease duration was 22 years), showed that complement deposits can be found consistently in areas of demyelination across all patients with established MS lesions. The authors suggested that the previously reported heterogeneity of white matter lesions (Lucchinetti et al., 2000) may have been specific to early disease and was therefore not seen in their autopsy samples. Subsequent immunohistochemical studies analyzing the presence and localization of key complement components (C3, factor B, C1q), activation products (C3b, iC3b, C4d, TCC/MAC), regulators (factor H, C1-inhibitor, clusterin) and receptors (C3aR, C5aR1) in established MS lesions found that, although variable between individuals, the presence of complement proteins and activation products in and around white matter lesions is a consistent feature of progressive MS (Ingram et al., 2014; Loveless et al., 2018), echoing the conclusions from Breij et al. (2008).

Evidence for complement activation in MS gray matter has also been mixed. Initial histological studies reported evidence of activated complement (C4d) on oligodendrocytes at the edge of small cortical plaques (Schwab and McGeer, 2002). Subsequent studies in autopsy tissue from MS patients with the long-standing disease showed little evidence for complement activation products in purely cortical lesions (Brink et al., 2005). More recent studies in lesions from progressive MS patients showed evidence of C1q deposition on neurons, as well as complement activation fragments (Bb, C3b) on neurons and glia, and TCC/MAC on neurons across cases analyzed (Watkins et al., 2016).

Intriguingly, complement may also play a role in the context of compartmentalized inflammation within meningeal TLT (see “MS Gray Matter” section; Ramaglia et al., in press a). Using the Th17 cell A/T model to induce EAE in SJL/J mice, Pikor et al. (2015) demonstrated that at the earliest stages of CNS autoimmunity, meningeal TLTs contain cells that are positive for the complement component C3. Although the nature of the C3^+^ cells within TLTs was not explored, deposited C3 could modulate adaptive immune cell responses. For example, activated C3 (C3d) bound to antigens has been shown to regulate B cell function by lowering the threshold for B cells activation through its interaction with the CR2 co-receptor on B cells (Dempsey et al., 1996). In terms of T cells, intracellular expression and activation of C3 have been implicated in the control of human adaptive T cell responses (Liszewski et al., 2013). One hypothesis is that C3a-like and C3b-like products are continuously generated intracellularly within endosomal/lysosomal compartments at low levels in resting T cells *via* cathepsin-L (CTSL)-mediated cleavage of C3; the C3a-like product engages the lysosomal-expressed G-protein-coupled C3a receptor (C3aR) to sustain the tonic activity of mammalian target of rapamycin complex 1 (mTORC1) and survival of circulating T cells at steady-state (Liszewski et al., 2013). The CTSL-mediated activation of C3 is increased by T cell receptor (TCR) activation and CD28 co-stimulation. C3 activation fragments then shuttle to the cell surface where they engage their respective receptors, C3aR (which binds C3a) and CD46 (which binds C3b), to induce IFN-γ production and T helper type 1 (Th1) differentiation (Liszewski et al., 2013). The same pathway could function in human CD8^+^ T lymphocytes. According to this hypothesis, C3 protein is processed intracellularly into activation fragments that engage the same receptors in an autocrine/paracrine manner and drives IFN-γ production and cytolytic activity (Arbore et al., 2018).

In line with the potential production and secretion of complement proteins by immune cells residing in meningeal TLTs, a recent proteomic CSF profiling in early MS patients showed that among 227 proteins differentially expressed between the patients with high vs. low cortical lesion load, 30% were related to the complement cascade, suggesting, that in addition to other soluble mediators, complement positively correlates with cortical damage at early disease stages (Magliozzi et al., 2019), possibly by diffusing through a disrupted glial limitans into the subpial cortex causing injury. Other proteins identified in the CSF of MS patients included the blood coagulation factor fibrinogen (Magliozzi et al., 2019), suggesting that the extrinsic pathway of the coagulation system may also be involved in pathological processes occurring in the MS brain. From the earliest studies using experimental animal models of demyelination, particularly EAE, it was already noted that perivascular deposition of insoluble fibrin [produced from fibrinogen by perivascular tissue factor and procoagulant proteins (Thomas et al., 1993; Akassoglou and Strickland, 2002)], occurred in conjunction with paralytic episodes (Paterson, 1976). Further studies causing abnormal cleavage and degradation of fibrinogen (Adams et al., 2004) or blocking the conversion of fibrinogen into insoluble fibrin (Akassoglou et al., 2004; Yang et al., 2011), reduced EAE severity. Experimental studies into the mechanisms of fibrin-induced injury performed by genetically blocking the ability of fibrin to bind the CD11b/CD18 integrin receptor on microglia and macrophages (without affecting the binding of other ligands to CD11b/CD18), resulted in reduced microglial activation, decreased axonal damage, less demyelination, and reduced paralysis, demonstrating that fibrinogen entry into the CNS and subsequent CD11b-mediated microglial activation may be key upstream molecular events that drive inflammatory demyelination (Adams et al., 2007; Davalos et al., 2012). More recently, it was shown that fibrinogen stored in extracellular vesicles from blood plasma induces encephalitogenic CD8^+^ T cells, contributing to the perpetuation of neuroinflammation and relapses in response to immunization with a myelin antigen (MOG_35–55_; Willis et al., 2019).

Indications that complement plays a role in the pathology of MS comes from experimental studies in animal models of demyelination, particularly EAE. From initial studies that have used cobra venom factor as a tool to consume complement, to subsequent studies that have used either untargeted or targeted pharmacological inhibition of complement activation or that have used genetic deletion of complement components and regulators, it has become apparent that reducing complement activation in EAE is protective (reviewed in Ingram et al., 2009). In particular, the “outside-in” role for systemically-derived complement in models of demyelination comes from recent evidence in the chronic relapsing EAE (crEAE) model of demyelination in Biozzi AB/H mice (Michailidou et al., 2018). In these mice, immunization with homogenates of the syngeneic spinal cord (SCH) in complete Freund’s adjuvant induces a chronic relapsing form of EAE (Baker et al., 1990), which pathologically is characterized by inflammatory demyelinating lesions in the spinal cord and substantial axonal injury (Jackson et al., 2009). In terms of inflammation, these spinal cord lesions are populated by macrophages, CD4^+^ T lymphocytes (Butter et al., 1991), and deposits of TCC/MAC (Ramaglia et al., 2015). Notably, post-symptomatic treatment of crEAE with an antisense oligonucleotide that specifically targets CNS-extrinsic production of murine C6 mRNA (a component necessary for the formation of the TCC/MAC complex), inhibited TCC/MAC deposition inside the CNS, prevented relapses and protected from relapse-induced axonal and synaptic damage (Michailidou et al., 2018). Further analysis showed that this protection was achieved by impeding the activation of parenchymal neuroinflammatory responses, including the Nod-like receptor protein 3 (NLRP3) inflammasome (Michailidou et al., 2018). Therefore antisense-mediated knockdown of C6 expression outside the CNS is sufficient to impede neuroinflammation in the crEAE model of MS, pointing to a pathogenic role of the serum-derived terminal complement components in this model of demyelination.

### Evidence Supporting the “Inside-Out” Paradigm

**Figure 5:**
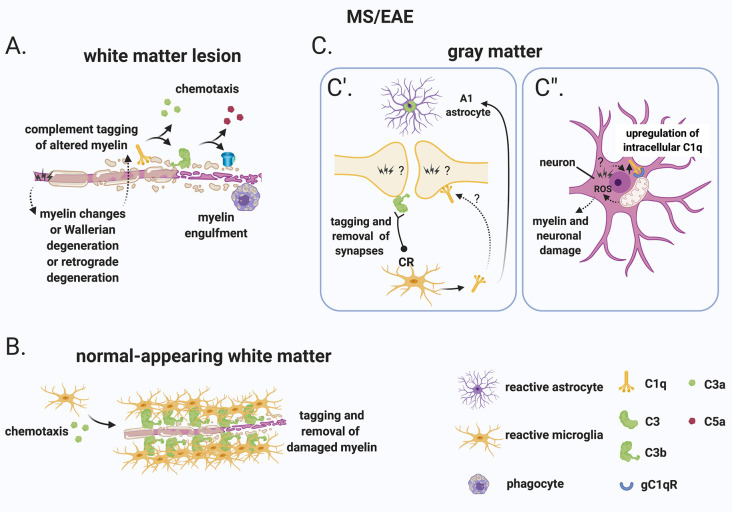
A role for complement in the “inside-out” paradigm of demyelination and synapse loss in MS.** (A)** The “inside-out” model argues that a so far unknown primary cytodegenerative event leads to myelin changes or Wallerian degeneration or retrograde degeneration which exposes altered highly antigenic myelin epitopes. In this scenario, the complement system would participate in the secondary autoimmune and inflammatory response by tagging myelin and promoting its clearance by phagocytes. **(B)** In the normal-appearing white matter, complement (particularly activated C3) tags axons that may be damaged as a result of Wallerian degeneration or retrograde degeneration. C3-tagged altered myelin is then removed by clusters of microglia (also called microglial nodules). **(C)** In the gray matter, neurons and synapses could be potential hotspots of complement-mediated primary cytodegeneration. **(C’)** Synapses (of the visual thalamus) are tagged by C3b and engulfed by microglia. Tagging of synapses by C1q has also been reported in the MS hippocampus but its role in the early engulfment of synapses has not yet been investigated. Also, microglia-derived C1q (together with IL-1α and TNF) induces a subtype of C3^+^ reactive astrocytes, termed A1 astrocytes, which are neurotoxic. **(C”)** Neurons upregulate C1q by an unknown trigger. C1q can then potentially bind to its receptor (gC1qR) on the surface of mitochondria triggering the production of ROS which in turn can induce myelin and neuronal damage. Abbreviations: ROS, reactive oxygen species.

Evidence that complement may be triggered as a reaction to primary damage within the CNS derives from studies in models of traumatic nerve or CNS injury. In these models, the primary injury is a mechanical insult either delivered by a compression of the sciatic nerve, inducing Wallerian degeneration (Ramaglia et al., 2007) or by a weight drop on a closed skull in the case of a traumatic brain injury (TBI; Fluiter et al., 2014) or on the spinal cord in the case of a spinal cord injury (SCI; Qiao et al., 2010). In each instance, the mechanical injury results in the activation of the complement system to completion including the formation of TCC/MAC (Anderson et al., 2004; Ramaglia et al., 2007; Nguyen et al., 2008; Fluiter et al., 2014). Importantly, inhibition of the complement system at various levels of the cascade results in reduced pathology and ameliorated clinical outcome in rodent models of nerve/CNS injury. For example, broad inhibition of the complement system by targeting the C3 convertase reduces neutrophil accumulation (Kaczorowski et al., 1995), promotes neuronal survival, induces neuroprotective intracerebral gene expression, and ameliorates neurological outcome after TBI (Leinhase et al., 2006). More specific inhibition of the complement cascade targeting only the alternative pathway of complement also led to substantial attenuation of cerebral tissue damage and neuronal cell death, inducing a neuroprotective pattern of intracerebral gene expression following TBI (Leinhase et al., 2007). Alternative strategies that have targeted specific pro-inflammatory arms of the complement system, such as inhibiting the anaphylatoxin C5a, have also shown protective effects by reducing neutrophil extravasation into the brain parenchyma after TBI (Sewell et al., 2004). Inhibition of the most downstream component of the terminal complement activation pathway, the formation of the membrane attack complex (TCC/MAC), is also sufficient to prevent secondary neurologic damage and neurologic deficit after TBI (Fluiter et al., 2014; Ruseva et al., 2015). In the case of SCI, deletion of C1q resulted in greater locomotor recovery and better histological outcome compared to wild-type mice after a contusion injury of the spinal cord (Galvan et al., 2008), suggesting that initiation of the classical complement pathway *via* C1q is detrimental to recovery after SCI. Involvement of the classical pathway of complement in determining pathology after SCI was also confirmed by the protective effects of C1 esterase inhibitor (C1-INH) on pathology and function after traumatic SCI in the rat (Tei et al., 2008). Blocking activation of the complement system at its core by deleting C3 or *via* targeted inhibition of C3 activation to sites of C3 deposition (with CR2-Crry) significantly reduced necrosis, demyelination, and neutrophil infiltration, improving functional outcome after SCI in mice (Qiao et al., 2006). Also, deletion of the alternative pathway component Factor B or pharmacological inhibition of the alternative pathway with a monoclonal antibody against Factor B reduced tissue damage and demyelination, reduced inflammatory cell infiltrate, and improved functional recovery after SCI in mice (Qiao et al., 2010). On the contrary, deletion of the TCC/MAC complement regulator CD59a resulted in significantly increased tissue injury and impaired functional recovery compared to wild-type mice (Qiao et al., 2010), also implicating the terminal complement pathway in the nerve injury caused by spinal cord trauma. Likewise in the peripheral nerve, blocking the C3 convertase (Ramaglia et al., 2008) or the TCC/MAC (Ramaglia et al., 2007) reduces pathology and neurological symptoms after trauma, whereas deletion of CD59a exacerbates Wallerian degeneration (Ramaglia et al., 2009a). Interestingly in the peripheral nerve, inhibition of TCC/MAC also accelerates the regeneration of the nerve (Ramaglia et al., 2009b), further proving a detrimental role of complement activation during nerve damage. Therefore, complement can be activated by degenerating axons in the absence of antibodies, demonstrating a reaction to altered-self epitopes. In the context of MS, a primary injury to myelinated axons or axons undergoing Wallerian degeneration could trigger secondary complement activation (Figure 5A).

Additional evidence that CNS-intrinsic complement activation may occur independently of immune cell-mediated demyelination comes from observations in the normal-appearing white matter or periplaque of MS brains. These regions are often reported to contain clusters of microglia (also called microglial nodules) around damaged axons coated with complement deposits (C3d; Prineas et al., 2001; Barnett et al., 2009; Ramaglia et al., 2012). While these C3d-associated microglial nodules have been proposed to play a role at the earliest stage of lesion formation (Marik et al., 2007; Barnett et al., 2009), a more in-depth analysis showed that, although nodules localize on axons with impaired transport, they likely do not reflect an acute attack against myelinated axons because they occur in chronic but not an acute disease and because they can also be detected in the brains of patients with non-demyelinating diseases such as ischemic stroke (Michailidou et al., 2017). Therefore, it was concluded that it is unlikely that microglial nodules around C3d-coated axons drive the formation of new lesions but could represent a physiological mechanism to remove irreversibly damaged axons in chronic disease (Michailidou et al., 2017; Figure 5B).

In the gray matter, neurons and synapses could be potential hotspots of complement-mediated primary cytodegeneration (Figure 5C). Spurred by evidence implicating early complement components in the pruning of synapses during development (Stevens et al., 2007; Schafer et al., 2012), adulthood (Wang et al., 2020), normal aging (Stephan et al., 2013; see “Early Complement Components in the Developing, Adult, and Normal Aging CNS” section) and neurodegeneration (Hong et al., 2016; see also “Early Complement Components in Neurodegeneration” section), studies of early complement components in the myelinated and demyelinated MS hippocampus were performed; these showed that C1q and C3d are deposited at synapses that localize within microglial processes and lysosomes particularly in the CA2/CA3 hippocampal subfield, supporting a role for complement-mediated elimination and degradation of synapses by microglia (Michailidou et al., 2015). A more recent study showed that in the MOG_35–55_ EAE mouse model of demyelination synapses are reduced while C1q and C3 are both upregulated in the CA1 hippocampal subfield at 28–30 days post-immunization. Notably, C3 deletion rescued synapse loss and improved clinical outcomes (Hammond et al., 2020). However, since complement C3 plays a broader role in the dynamics of EAE, it is difficult to ascribe the protective effects of C3 deletion to a local role of C3 within the hippocampus. Studies at early (pre-onset) stages of EAE or in non-immune mediated models of hippocampal demyelination (such as the cuprizone model) may be more informative at pinpointing the kinetics of complement-dependent pruning of synapses in MS. Indeed, a recent study analyzing brain tissue at the onset of moderate clinical symptoms of models of demyelination showed that synaptic material is tagged by C3 (but not by C1q) and is engulfed by microglia in the retinogeniculate system of models of demyelination (Werneburg et al., 2020), dissociating complement activation at synapses from immune-mediated demyelination in this model (Figure 5C’).

What initiates complement activation in the MS hippocampus and visual thalamus is unknown but is an important question. The observations that neuronal production of C1q has been observed in the MS hippocampus (Michailidou et al., 2015) and in the retinogeniculate system (Bialas and Stevens, 2013) would suggest that the signal to eliminate synapses originates from the target neuron. The mechanisms responsible for the recruitment of C1q to synapses also remains unknown. Antibodies are the obvious candidate, but deposits of immunoglobulins were not detected in the MS hippocampus. An alternative target of C1q is an apoptotic signal on the surface of dead or dying cells, such as the previously identified ceramide transporter protein (Bode et al., 2014) or the externalization of phosphatidylserine (PS; Scott-Hewitt et al., 2020). Exposed PS have been shown in the cortex of adult mice in isolated synaptosomes that are also tagged by C1q (Györffy et al., 2018) and have also been observed *in vivo* at presynaptic inputs in the hippocampus and in the dLGN of developing mice (Scott-Hewitt et al., 2020). During development, deletion of C1q results in increased PS-labeled presynaptic inputs and decreased microglial engulfment of PS-labeled elements (Scott-Hewitt et al., 2020), implicating C1q in the elimination of PS-labeled synapses. It is likely that in this context, PS-labeled elements are engulfed by microglia *via* the phagocytic receptor TREM2 since, in hippocampal neuron and microglia co-cultures, synapse elimination can be partially prevented by deleting TREM2 on microglia (Scott-Hewitt et al., 2020). While this evidence point towards a potential role for an apoptotic signal in the C1q-mediated elimination of synapses which could be of significance in the MS brain, MS neurons are quite resistant to cell death (Dutta et al., 2011) which argues against C1q-driven opsonization of apoptotic neurons. Notably, local pruning of dendritic spines can occur by a caspase-3–dependent mechanism without inducing apoptosis (Ertürk et al., 2014). This evidence suggests that programmed cell death may be initiated but spatially restricted by inhibitors that allow the elimination of synapses without killing the target neuron. This pathway of local pruning of dendrites is initiated by the mitochondrial production of reactive oxygen species (ROS) and/or by the activation of the N-methyl-D-aspartate receptor (NMDA) receptors locally in dendrites (Ertürk et al., 2014). Both mitochondrial oxidative stress (Mahad et al., 2009; Witte et al., 2009; Fischer et al., 2012) and changes in the glutamate neurotransmitter system (Dutta et al., 2011) have been reported in MS. Based on these findings it is tempting to speculate that similar pathways may regulate synaptic pruning in the MS hippocampus. Also, poor tissue oxygenation (hypoxia) and “virtual hypoxia” could cause axonal changes that could potentially be targeted by the complement system. Hypoxia could arise partly from narrow-end arteries and arterioles that are vulnerable to oxygen desaturation or from veins that can deplete oxygen from the surrounding tissue (Martinez Sosa and Smith, 2017). “Virtual hypoxia” could arise from increased energy demand of demyelinated axons to generate action potentials and reduced axonal ATP production (Trapp and Stys, 2009).

In the context of the “inside-out” paradigm, a potential mechanism that has been suggested as a trigger of cytodegeneration is a putative deficiency of copper ions in the MS-affected CNS, with copper deficiency promoting demyelination and loss of myelinating oligodendroglia. This hypothesis originates from observations in the cuprizone model of demyelination, where the copper chelator cuprizone, results in varying degrees of oligodendroglial damage and demyelination in the CNS, with limited inflammation (Kipp et al., 2009). Since copper ions are known to desensitize NMDA receptors (Stys et al., 2012a; You et al., 2012), and in addition to neurons, oligodendrocytes and the myelin sheath also express NMDARs (Karadottir et al., 2005; Micu et al., 2006), it was suggested that dysregulation of copper homeostasis may result in chronic overactivation of these receptors, leading to excitotoxicity (Stys et al., 2012b). In this setting, it is tempting to speculate that the elimination of synapses by complement may be initially a protective mechanism that lowers excessive excitatory activity since such excitatory activity can eventually lead to pathological synaptic alterations. This hypothesis, however, remains to be tested.

## Intracellular C1q in the CNS—“Inside the Inside”

As mentioned, C1q is at least partially produced by cortical neurons in MS (Michailidou et al., 2015; Watkins et al., 2016) and neuronal production of C1q has also been observed in the retinogeniculate system (Bialas and Stevens, 2013). Intracellular C1q is emerging as an important modulator of cell metabolism particularly by acting on mitochondrial activity. For example, intracellular C1q can be recognized by the mitochondrially expressed receptor gC1qR (Dedio et al., 1998) and was shown to drive mitochondrial production of ROS and subsequent neuronal death during hypoxia-mediated damage (Ten et al., 2010), which have been shown to occur in MS lesions (Mahad et al., 2009; Trapp and Stys, 2009; Witte et al., 2009; Fischer et al., 2012; Martinez Sosa and Smith, 2017).

ROS are chemically reactive species that can mediate demyelination and neurodegeneration in the MS-affected CNS. While in MS lesions the primary source of oxygen and nitric oxide radicals has been attributed to the oxidative burst induced in activated microglia and macrophages in the course of inflammation (Liu et al., 2001; Marik et al., 2007; Gray et al., 2008; Zeis et al., 2009; Fischer et al., 2012, 2013; Zrzavy et al., 2017), it remains possible that ROS also originates within neurons. Evidence of oxidative injury has been found in active MS lesions and includes oxidized lipids and proteins, as well as nitrosylated epitopes (Vladimirova et al., 1998; Bizzozero et al., 2005; Zeis et al., 2009). This is particularly evident in degenerating neurons and axons as well as in oligodendrocytes dying by apoptosis (Haider et al., 2011). Oxygen and nitric oxide radicals can also induce mitochondrial injury by disrupting mitochondrial enzyme function, by modifying mitochondrial proteins and accelerating their degradation. Free radicals can also interfere with *de novo* synthesis of respiratory chain components and can directly induce mitochondrial DNA damage (Bolaños et al., 1997; Smith et al., 1999). Therefore, the role of intracellular C1q in driving the production of ROS is consistent with the evidence of ROS and ROS-mediated injury in MS tissue.

In addition to C1q, C1q-TNF family proteins [also known as C1q-TNF-related protein (CTRP) family] play many roles in both immunity and metabolism. These molecules are structurally related to both C1q and TNF and form hybrid proteins (recently reviewed in Schaffler and Buechler, 2012). The C1q-TNF-CTRP family member, CTRP3, negatively regulates lipid metabolism by downregulating PPAR-γ and C/EBPα in adipocytes (Nishimoto et al., 2017), it has also been shown to mediate mitochondrial ROS production in smooth muscle cells (Feng et al., 2016), and it protects mesenchymal stem cells from ischemia-induced apoptosis *via* activation of the PI3K/Akt pathway (Hou et al., 2014). Importantly, CTRP3 knockdown by siRNA in bone marrow-derived mesenchymal stem cells proved that intracellular and/or autocrine C1q synthesis rather than systemic production was important for its function (Hou et al., 2014). While altered lipid metabolism (Kooij et al., 2019), mitochondrial ROS production (see above), and apoptosis (Lucchinetti et al., 2000) occur in MS, whether intracellular C1q plays a role in the ROS-mediated damage observed in the MS-affected CNS remains to be investigated (Figure 5C”).

## Approaches to Therapies Targeting the Complement System in MS

Regardless of the modality in which the complement system is involved in MS, either as part of a primary autoimmune attack to the CNS (outside-in) or as part of a secondary event triggered by primary changes in the CNS (inside-out), uncontrolled activation can ultimately exacerbate an injury. Thus inhibition of complement may be protective. Identification of MS patients that may benefit from complement therapy, or pin-pointing a time-frame when complement therapy may be appropriate, would be invaluable in guiding clinicians to decide whether and when an anti-complement drug might be effective in a given patient. Measuring complement may also aid diagnosis, assessment of prognosis, and help in the monitoring of treatment response in this complex and heterogeneous disorder. Besides, although not reviewed here in detail, complement can also exert protective functions in the brain (reviewed in Ingram et al., 2009). Therefore, targeting complement-mediated injurious signals while maintaining potential protective roles, would have to be taken into account when designing an appropriate complement therapy.

Various complement proteins have also been considered as biomarkers of disease activity. One challenge in the identification of complement as a serological or CSF biomarker is the fact that complement components are acute-phase proteins whose synthesis and consumption are both increased in response to inflammation. As a consequence, the serum and CSF levels of a given complement component will be influenced by bouts of active inflammation, including infection. Nevertheless, while earlier studies showed inconsistent results (Jans et al., 1984; Jongen et al., 2000), more recent analysis on well-powered cohorts stratified based on clinical course and compared to age-matched controls have shown more consistent outcome for levels of complement components, activation products and regulators in serum and CSF (Ingram et al., 2010a,b, 2012). Systemic complement profiling has shown increased plasma levels of C3, C4, C4a, C1 inhibitor, and factor H, while levels of the terminal component C9 were reduced in MS patients compared with controls. Importantly, combined profiling of these analytes produced a statistical model with a predictive value of 97% for MS and 73% for clinical relapse when combined with selected demographic data (Ingram et al., 2012). Interestingly, plasma C4a levels were found to be raised only in acute relapse, decreasing over 2 months (Ingram et al., 2010b) and serum factor H levels were capable of distinguishing secondary progressive from relapsing-remitting disease (excluding patients in clinical relapse) with a sensitivity of 89.41%, a specificity of 69.47% and a positive predictive value of 72.38% (Ingram et al., 2010a). Therefore, specific complement components may be an effective indicator of progression or relapse and accessible biomarker to stratify patients, providing objective evidence to help guide therapeutic decisions.

While there is a growing complement therapeutics industry with many new emerging drugs (Carpanini et al., 2019), to date CNS targets have been largely ignored. Drug delivery across the BBB is a major challenge that needs to be considered when designing CNS-directed therapeutics. Perhaps therapies should target areas of pathology, as described for the fusion proteins linking CR2 (localizes to C3 activation products in tissues) with a complement regulator (see Werneburg et al., 2020), or they should target complexes, for example, TCC/MAC, which is found only in areas of pathology (reviewed in Morgan and Harris, 2015).

Although much remains to be clarified, targeting specific effector pathways of complement in (a subgroup of) patients at a crucial time(s) during the disease course could be a useful therapeutic approach in the future.

## Conclusions and Future Directions

In conclusion, while it is clear that the complement system is involved in the pathology of the MS-affected CNS, the pathways that the complement system engages to aid in the clearance of myelin or to shape synaptic circuits in various regions of the MS brain can differ substantially. Increasing awareness of the distinct roles of complement components in normal brain development and in neurodegenerative disorders may guide our understanding of similar pathological processes in MS. Understanding the relative contribution of serum-derived complement proteins vs. those produced locally by brain resident cells across MS disease stages and how to complement gene expression is regulated in the brain will be important questions for future research. For example, conditional knock out mice in which a particular complement gene can be inactivated in specific cell types of the brain could help tease apart the contributions of complement derived from brain-resident cells vs. that derived from the periphery. Also, viral vector approaches that enable the expression of complement regulators at targeted surfaces within the brain could help test the effect of local complement activation while maintaining intact peripheral complement functions. As a complementary approach, bone marrow chimeras or targeted inhibition of hepatic source of complement could help modulate peripheral complement while maintaining intact complement produced by cells within the brain. The added knowledge may help answer the question of whether MS is initiated by “outside-in” or “inside out” disease mechanisms. The current knowledge is equally consistent with either paradigm, and it is likely that both mechanisms jointly contribute to the variable course of MS.

## Author Contributions

VR, JLG and BPM wrote the manuscript. All authors contributed to the article and approved the submitted version.

## Conflict of Interest

VR is co-inventor of a patent that describes the use of inhibitors of the terminal complement pathway for therapeutic purposes; she is co-founders of Regenesance B.V. and received consulting honorarium from EMD Serono and Fluidigm. JLG has received funding from Roche, Novartis and EMD Serono for research and funding from Roche for consulting activities. BPM is a consultant for RaPharma.
